# Nomogram model based on preoperative clinical characteristics of unilateral papillary thyroid carcinoma to predict contralateral medium-volume central lymph node metastasis

**DOI:** 10.3389/fendo.2023.1271446

**Published:** 2024-02-12

**Authors:** Fan Wu, Kaiyuan Huang, Xuanwei Huang, Ting Pan, Yuanhui Li, Jingjing Shi, Jinwang Ding, Gang Pan, You Peng, Yueping Teng, Li Zhou, Dingcun Luo, Yu Zhang

**Affiliations:** ^1^ Department of Oncological Surgery, Affiliated Hangzhou First People’s Hospital, Westlake University School of Medicine, Hangzhou, Zhejiang, China; ^2^ The Fourth Clinical Medical College, Zhejiang Chinese Medical University, Hangzhou, Zhejiang, China; ^3^ Cancer Center, Department of Pathology, Zhejiang Provincial People's Hospital (Affiliated People's Hospital), Hangzhou Medical College, Hangzhou, Zhejiang, China; ^4^ Department of Head and Neck Surgery, Cancer Hospital of the University of Chinese Academy of Sciences, Hangzhou, China; ^5^ Operating Room, Affiliated Hangzhou First People’s Hospital, Westlake University School of Medicine, Hangzhou, Zhejiang, China

**Keywords:** papillary thyroid carcinoma, contralateral, central lymph node metastasis, plateletto-lymphocyte ratio, nomogram

## Abstract

**Objectives:**

To explore the preoperative high-risk clinical factors for contralateral medium-volume central lymph node metastasis (conMVCLNM) in unilateral papillary thyroid carcinoma (uPTC) and the indications for dissection of contralateral central lymph nodes (conCLN).

**Methods:**

Clinical and pathological data of 204 uPTC patients who underwent thyroid surgery at the Hangzhou First People’s Hospital from September 2010 to October 2022 were collected. Univariate and multivariate logistic regression analyses were conducted to determine the independent risk factors for contralateral central lymph node metastasis (conCLNM) and conMVCLNM in uPTC patients based on the preoperative clinical data. Predictive models for conCLNM and conMVCLNM were constructed using logistic regression analyses and validated using receiver operating characteristic (ROC) curves, concordance index (C-index), calibration curves, and decision curve analysis (DCA).

**Results:**

Univariate and multivariate logistic regression analyses showed that gender (P < 0.001), age (P < 0.001), tumor diameter (P < 0.001), and multifocality (P = 0.008) were independent risk factors for conCLNM in uPTC patients. Gender(P= 0.026), age (P = 0.010), platelet-to-lymphocyte ratio (PLR) (P =0.003), and tumor diameter (P = 0.036) were independent risk factors for conMVCLNM in uPTC patients. A predictive model was established to assess the risk of conCLNM and conMVCLNM, with ROC curve areas of 0.836 and 0.845, respectively. The C-index, the calibration curve, and DCA demonstrated that the model had good diagnostic value.

**Conclusion:**

Gender, age, tumor diameter, and multifocality are high-risk factors for conCLNM in uPTC patients. Gender, age, tumor diameter, and PLR are high-risk factors for conMVCLNM in uPTC patients, and preventive conCLN dissection should be performed.

## 1 Introduction

Thyroid papillary carcinoma (PTC) is the most common type of differentiated thyroid cancer. Central lymph node metastasis (CLNM) is very common in PTC patients (1), and it is one of the important factors affecting recurrence and survival (2, 3). Clinical practice guidelines (4–6) recommend prophylactic central lymph node dissection for unilateral papillary thyroid carcinoma (uPTC); however, at present, guidelines recommend only routine ipsilateral central lymph node dissection (CLND) (3, 7). Patients with uPTC may present with contralateral central lymph node metastasis (conCLNM), with an incidence ranging from 3.88% to 34.6% (8, 9). For these patients, if dissection is not performed, there may be a risk of recurrence, which can affect patient prognosis; however, routine dissection significantly increases the chances of parathyroid dysfunction, with a possibility of permanent hypoparathyroidism (10, 11), and nerve damage. Therefore, it is important to rule out conCLNM.

Stratification based on the diameter of metastatic lymph nodes can effectively predict the prognosis of patients. The 2015 American Thyroid Association(ATA) guidelines define small-volume lymph node metastasis as clinically node-negative (cN0) or ≤5 metastatic lymph nodes with a diameter of <0.2 cm, whereas the guidelines define large-volume lymph node metastasis as metastatic lymph nodes with a diameter of ≥3 cm. However, there is no accepted definition for lymph node metastasis types of > 5 lesions and a diameter of <3 cm or <5 lesions with a diameter of ≤0.2 cm. It has been reported that this type of lymph node metastasis, defined as medium-volume lymph node metastasis, significantly positively impacts disease the recurrence (12, 13). However, the recommendation based on existing diagnostic and therapeutic guidelines, which only suggest ipsilateral CLND, may miss metastatic lymph nodes in these patients.

Studies have reported that preoperative blood immune indicators, such as the neutrophil-to-lymphocyte ratio (NLR), lymphocyte-to-monocyte ratio (LMR), platelet-to-lymphocyte ratio (PLR), and systemic immuno-inflammatory index (SII), can be used to evaluate immune function, characterize tumors, and predict tumor progression (14, 15). It has been reported that NLR, PLR and MLR can have good sensitivity and accuracy in predicting lymph node metastasis in differentiated thyroid cancer (16). In addition, the increase of PLR is associated with lymph node metastasis (17).However, the correlation between blood immune indicators and conCLNM or contralateral medium central volume lymph node metastasis (conMVCLNM) in uPTC remains unclear. This study seeks to explore the relationship between the preoperative clinical features of uPTC patients with conCLNM or conMVCLNM to provide a basis for accurate diagnosis and personalized treatment.

## 2 Materials and methods

### 2.1 Study patients

A retrospective analysis of clinical data was conducted on 204 patients from September 2010 to October 2022 at Hangzhou First People’s Hospital. The patients were divided into two groups based on the presence of conCLNM (50 cases with conCLNM, and 154 cases without conCLNM) or conMVCLNM (26 cases with conMVCLNM, and 178 cases without conMVCLNM) (**Table 1**).

**Table 1:** General characteristics of PTC in conCLNM and conMVCLNM.

		Without conCLNM	With conCLNM	P value	Without conMVCLNM	With conMVCLNM	P value
All		154	50		178	26	
Gender	Male	17(11.0%)	24(48.0%)	<0.001	27(15.2%)	14(46.2%)	0.001
	Female	137(89.0%)	26(52.0%)		151(84.8%)	12(53.8%)	
Age	M(IQR)	51.000 (40.000,57.000)	35.000 (30.000,49.000)	<0.0001	49.000 (38.000,57.000)	35.000 (29.000,46.000)	<0.001
Tumor diameter(mm)	M(IQR)	9.000(5.000,15.000)	12(9.000,26.500)	<0.001	9.500 (6.000,15.000)	15.000 (9.000,30.000)	0.009
PLR	M(IQR)	129.060 (104.349,161.648)	97.772 (74.286,130.769)	0.002	127.071 (102.778,161.429)	84.526 (66.296,103.103)	<0.001
NLR	M(IQR)	1.720 (1.430,2.290)	1.570 (1.200,2.130)	0.272	1.720 (1.400,2.290)	1.340 (1.070,1.770)	0.108
MLR	M(IQR)	0.180 (0.140,0.250)	0.165 (0.130,0.230)	0.257	0.180 (0.140,0.250)	0.140 (0.130,0.250)	0.356
SII	M(IQR)	391.925 (289.710,509.440)	335.600 (248.750,476.860)	0.238	391.925 (282.180,509.440)	310.745 (239.480,388.270)	0.073
Multifocality	Yes	23(14.9%)	17(34.0%)	0.003	32(18.0%)	8(30.8%)	0.125
	No	131(85.1%)	33(66.0%)		146(82.0%)	18(69.2%)	
Capsule invasion	Yes	61(39.6%)	31(62.0%)	0.006	77(43.3%)	15(57.7%)	0.167
	No	93(60.4%)	19(38.0%)		101(56.7%)	11(42.3%)	
Extrathyroidal extension	Yes	42(27.3%)	20(40.0%)	0.089	52(29.2%)	10(38.5%)	0.338
	No	112(72.7%)	30(60.0%)		126(70.8%)	16(61.5%)	
PTMC	Yes	64(41.6%)	29(58.0%)	0.043	78(43.8%)	15(57.7%)	0.185
	No	90(58.4%)	21(42.0%)		100(56.2%)	11(42.3%)	
Lesion location	Down	45(29.2%)	20(40.0%)	0.155	51(28.7%)	14(53.8%)	0.010
	Non-down	109(70.8%)	30(60.0%)		127(71.3%)	12(46.2%)	

conCLNM, contralateral central lymph node metastasis; conMVCLNM, contralateral medium-volume central lymph node metastasis; IQR, interquartile range; LMR, lymphocyte-to-monocyte ratio; M, median; NLR, neutrophil-to-lymphocyte ratio; PTMC, papillary thyroid microcarcinoma; PLR, platelet-to-lymphocyte ratio; SII, preoperative systemic immune-inflammatory index.

### 2.2 Inclusion and exclusion criteria

The inclusion criteria were as follows: (1) patients who were postoperatively diagnosed with unilateral papillary thyroid carcinoma, (2) patients who underwent at least total thyroidectomy and bilateral central lymph node dissection, and (3) patients with complete clinical and pathological information. The exclusion criteria were as follows: (1) patients with pathological evidence of malignant PTC which was not treated by lymph node dissection, (2) patients with hyperthyroidism or those that underwent previous history of thyroid radiotherapy, or thyroid surgery, (3) patients with previous history of other malignancies, and (4) patients with hematological disorders, autoimmune disorders, acute or chronic inflammatory diseases, or other diseases that may affect routine blood tests. This study was approved by the Ethics Committee of Hangzhou First People’s Hospital, and written informed consent was obtained from all patients.

### 2.3 Data acquisition

Peripheral blood was obtained from the patients one week prior to thyroid surgery and processed using the automated Mindary BC-6800 blood cell analyzer (Shenzhen Mairui Biomedical Electronics Co., Ltd., Shenzhen, China) along with appropriate reagents. Peripheral blood cells were classified and counted using a combination of a sheath flow impedance method, a laser light scattering method, and flow cytometry coupled with fluorescent staining techniques. The preoperative inflammatory indicators were included preoperative platelet count, neutrophil count, lymphocyte count, macrophage count, PLR, NLR,LMR, and platelet count × neutrophil count/lymphocyte count (SII).

The diagnostic apparatuses used for thyroid examinations were the Mylab 70 XVG and MyLab Twice Color Doppler ultrasound systems, with a probe frequency of 7 to 13 MHz. The instrument settings were optimized for this purpose. Patients were placed in a supine position to allow for full neck exposure. Upon identification of a suspicious malignant nodule, the two-dimensional ultrasound image features of the nodule were interpreted and recorded. Two experienced ultrasound physicians assessed the preoperative tumor information by ultrasonography, including tumor diameter, capsule invasion, extrathyroidal extension, multifocality, and lesion location.

**Figure 1:**
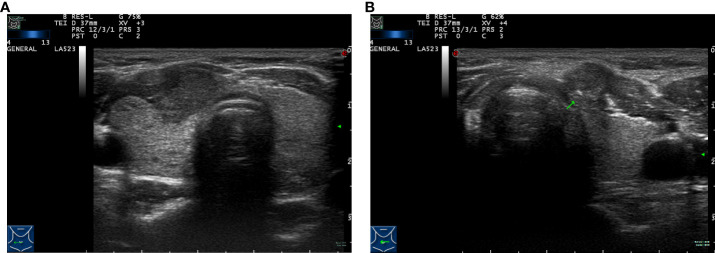
Preoperative ultrasound images of tumor lesions. **(A)** Capsule invasion. **(B)** Extrathyroidal extension (arrow).

**Figure 2:**
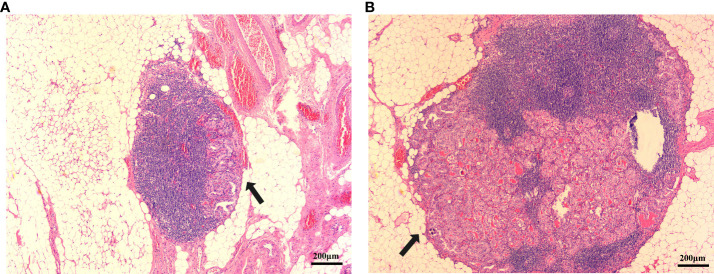
PTC metastatic lymph node lesion diameter. **(A)** Metastatic lymph node lesion diameter is <0.2 cm(arrow). **(B)** Metastatic lymph node lesion diameter is >0.2 cm. (hematoxylin and eosin; ×40) (arrow).

The ultrasound images included in this study were reviewed and preserved by two senior physicians who were part of the same team. They were also evaluated by the chief sonographer. Simultaneously, all members reading the ultrasounds blinded to the final pathology results. The diagnostic criterion for capsular invasion was the disappearance of the echogenic thyroid capsule at the site of contact with the thyroid cancer on ultrasound examination (**Figure 1A**). The diagnostic criterion criteria for extrathyroidal invasion was destruction or the invasion of adjacent structures at the edge of the thyroid capsule on ultrasound examination (**Figure 1B**). The ultrasound evaluation criteria of lymph node metastasis (one or more of the following high-risk factors are considered lymph node metastasis) were as follows: 1)focal or diffuse hypoechogenicity; 2) ratio of the short axis to long axis ≥0.5; 3)lymphatic echogenic hilus absence; 4) cystic degeneration; 5) microcalcifications; and 6) chaotic vascular flow (18–20). The presence of lymph node metastasis and the maximum diameter of the metastatic foci in the lymph nodes were accurately determined by two pathologists with clinical research experience by microscopy (**Figure 2**).

### 2.4 Statistical analyses

Data were analyzed using SPSS software (version 25.0; IBM, Armonk, NY, USA). For measurement data, we conducted the normality test and presented the results as mean ± standard deviation (± S). Nonconforming data was expressed as the medians and interquartile ranges. To compare two independent groups, we used the Student’s t-test. Categorical data were expressed as frequency and percentage, and group comparisons were performed using the chi-square (X^2^) test. By utilizing univariate analyses, we identified the risk factors associated with conCLNM/conMVCLNM in PTC patients by pinpointing the influencing factors that exhibited statistically significant differences.

We utilized logistic multivariate stepwise regression analysis to examine the risk factors identified through univariate regression analysis. In addition, we analyzed the differences in preoperative blood inflammatory indexes and ultrasound features of conCLNM/conMVCLNM and identified the independent risk factors that impact conCLNM/conMVCLNM in PTC patients. We determined the odds ratios (OR) and 95% confidence intervals (95% CI).

Based on the results of logistic multivariate regression analysis, we constructed a predictive model. Receiver operator characteristic (ROC) curve analysis was employed to assess the area under the curve (AUC) and its 95% CI. We calculated the Youden index and identified the sensitivity and specificity at its highest value. To validate the predictive model, we conducted validation through bootstrap sampling. We evaluated the models using the AUC, 95% CI, sensitivity, and specificity. Additionally, we created a calibration plot. Decision curve analysis (DCA) was employed to validate the clinical net benefit rate of the predictive model. The statistical analysis was performed using R studio (version 4.0.2). We considered P < 0.05 as statistically significant.

## 3 Results

### 3.1 Clinical characteristics and basic information

According to the inclusion and exclusion criteria, a total of 204patients were enrolled. In the conCLNM group, among the 50 patients with conCLNM, there were 24 males (48.0%) and 26 females (52.0%), with a mean age of 39.400 ± 15.534 years. In conMVCLNM group, among the 26 patients with conMVCLNM, there were 14 males (53.8%) and 12 females (46.2%), with a mean age of 37.692 ± 17.022 years. The general characteristics of the patients are shown in **Table 1**.

### 3.2 Univariate and multivariate analyses for preoperative conCLNM and conMVCLNM variables

In the conCLNM group, univariate analysis showed that gender, age, preoperative PLR, papillary thyroid microcarcinoma (PTMC), multifocality, capsule invasion, and maximum tumor diameter were risk factors for conCLNM. Multivariable logistic regression analysis showed that gender (P < 0.001, OR: 7.547, 95% CI: 3.227–17.653), age (P < 0.001, OR: 0.952, 95% CI: 0.925–0.980), tumor diameter (P < 0.001, OR: 1.068, 95% CI: 1.027–1.110), and multifocality (P = 0.008, OR: 3.375, 95% CI: 1.383–8.232) were independent risk factors for conCLNM. In the conMVCLNM group, univariate analysis showed that gender, age, preoperative PLR, lesion location and tumor diameter were significantly correlated with conMVCLNM (P < 0.05). Multivariable logistic regression analysis showed that gender (P = 0.026, OR: 3.251, 95% CI: 1.148–9.204), age (P = 0.010, OR: 0.954, 95% CI: 0.921–0.982), preoperative PLR (P = 0.003, OR: 0.977, 95% CI: 0.962–0.992), and tumor diameter (P = 0.036, OR: 1.049, 95% CI: 1.003–1.096) were independent risk factors for conMVCLNM (**Table 2**).

**Table 2:** Univariate logistic regression analysis of uPTC in conCLNM and conMVCLNM.

	B	SE	Wals	Sig.	Exp (B)	95% CI
conCLNM						
Gender	2.021	0.434	21.736	<0.001	7.547	3.227–17.653
Age	-0.049	0.015	11.729	<0.001	0.952	0.925–0.980
Tumor diameter (mm)	0.065	0.020	10.724	<0.001	1.068	1.027–1.110
Multifocality	1.216	0.455	7.146	0.008	3.375	1.383–8.232
conMVCLNM						
Gender	1.179	0.531	4.929	0.026	3.251	1.148–9.204
Age	-0.047	0.018	6.668	0.010	0.954	0.921–0.982
PLR	-0.023	0.008	8.603	0.003	0.977	0.962–0.992
Tumor diameter (mm)	0.048	0.023	4.404	0.036	1.049	1.003–1.096

B, Beta coefficient; CI, Confidence interval; conCLNM, contralateral central lymph node metastasis; conMVCLNM, contralateral medium-volume central lymph node metastasis; PLR, platelet-to-lymphocyte ratio; SE, Standard error of the mean; Sig, Statistical significance.

### 3.3 Construction of the nomogram

**Figure 3:**
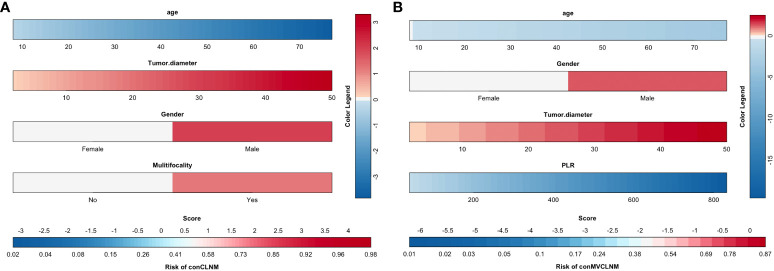
Clinical prediction models for conCLNM and conMVCLNM. Prediction model based on clinical factors **(A)** conCLNM and **(B)** conMVCLNM. conCLNM, contralateral central lymph node metastasis; conMVCLNM, contralateral medium volume central lymph node metastasis; uPTC, unilateral papillary thyroid carcinoma; PLR, platelet-to-lymphocyte ratio.

The results screened by multivariable logistic regression analysis were incorporated into the prediction model for distinguishing conCLNM and conMVCLNM in uPTC patients (**Figure 3**). The score for each independent predictive factor was plotted and summed continuously to obtain a total score, which determined the likelihood of developing conCLNM or conMVCLNM.

### 3.4 Evaluation of the nomogram

**Figure 4:**
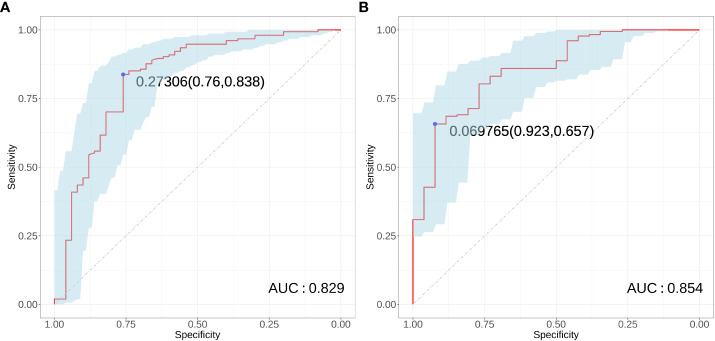
Receiver operator characteristic curve for conCLNM and conMVCLNM. Receiver operating characteristic curves **(A)** conCLNM and **(B)** conMVCLNM. conCLNM, contralateral central lymph node metastasis; conMVCLNM, contralateral medium volume central lymph node metastasis; uPTC, unilateral papillary thyroid carcinoma; ROC, receiver operator characteristic.

The results of ROC curve analysis showed that the AUCs of the four risk factors (gender, age, tumor diameter, and multifocality) in predicting conCLNM in uPTC patients was 0.829 (P < 0.001, 95% CI: 0.756–0.902). When the Youden index was highest, the specificity was 76.0% and the sensitivity was 83.8%, indicating good discrimination (**Figure 4A**). The AUCs of the four risk factors (age, gender, PLR and tumor diameter) in predicting conMVLNM was 0.854 (P < 0.001, 95% CI: 0.780–0.929). When the Youden index was highest, the specificity was 65.7% and the sensitivity was 92.3% (**Figure 4B**).

**Figure 5:**
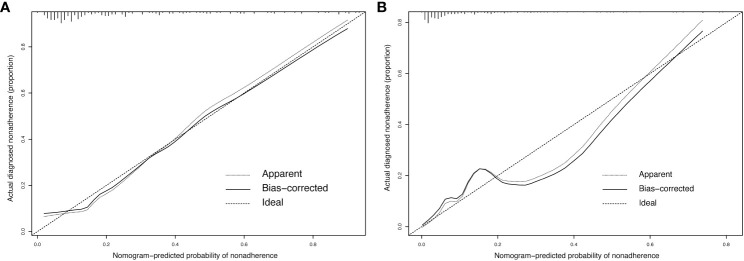
Calibration curve for conCLNM and conMVCLNM. Calibration curve **(A)** conCLNM and **(B)** conMVCLNM. The X-axis represents the predicted probability of conCLNM or conMVCLNM, whereas the Y-axis represents the actual probability of diagnosed conCLNM and conMVCLNM. The diagonal dashed line represents the ideal prediction model. The solid line represents the performance of the column chart model, with a higher fit to the diagonal dashed line indicating better predictive capability. conCLNM, contralateral central lymph node metastasis; conMVCLNM, contralateral medium volume central lymph node metastasis.

The original dataset was resampled 1000 times using bootstrapping to establish the simulation dataset. The calibration curve demonstrated good consistency between the discrimination of the prediction model and the actual distinction between conCLNM and conMVCLNM. The mean absolute errors in the calibration curve were 0.02 and 0.055 for conCLNM and conMVCLNM, respectively, indicating good consistency between the predicted and actual values (**Figure 5**).

**Figure 6:**
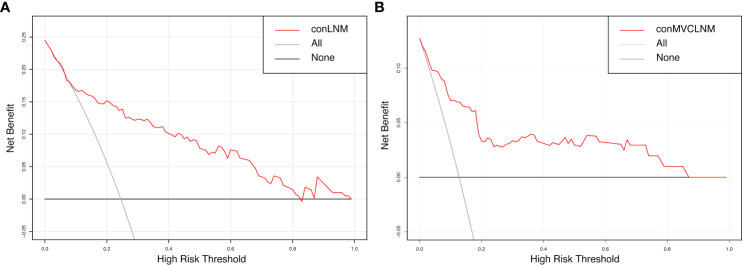
Decision curve analysis for conCLNM and conMVCLNM. Decision curve analysis **(A)** conCLNM and **(B)** conMVCLNM. The red line represents the prediction model. The grey line represents the scenario where all patients are classified as conCLNM or conMVCLNM positive. The horizontal black line represents the scenario where all patients are classified as conCLNM/conMVCLNM negative. conCLNM, contralateral central lymph node metastasis; conMVCLNM, contralateral medium volume central lymph node metastasis.

The normal range of the concordance index (C-index) is typically between 0.5 and 1, with 0.5 indicating a model with poor discrimination and 1 indicating a model with perfect discrimination. A C-index >0.7 indicates a model with strong discrimination. The C-indices for conCLNM and conMVCLNM were 0.836 and 0.845, respectively, indicating models with excellent discrimination. DCA demonstrated the effectiveness of the nomograms for both groups across a wide range of threshold probabilities (**Figure 6**).

## 4 Discussion

Our study reports that age, preoperative PLR, tumor diameter, and lesion location are independent risk factors for conMVCLNM in uPTC patients. Our model effectively identifies conMVCLNM and avoids missed diagnosis or overdiagnosis to achieve the purpose of accurate diagnosis and treatment.

Based on the results of this study, we found that clinical features can effectively predict conCLNM or conMVCLNM. Other studies have reached similar conclusions; for example, a meta-analysis by Sun et al. (21) showed that gender, age, and extrathyroidal extension (ETE) are risk factors for conCLNM in uPTC. At the same time, another investigation has found that postoperative pathological features, such as multifocality and tumor diameter, are independent risk factors for conCLNM (22). However, all of these studies were retrospective in nature, and they only screened high-risk factors from postoperative pathology and failed to provide effective guidance before surgery, which needs to be improved in terms of the clinical application. In our study, the risk of conCLNM in uPTC could be predicted by preoperative indicators, and this approach is clinically practical. Additionally, in the prospective study by Chen et al. (8), bilateral CLND is recommended when preoperative imaging or intraoperative frozen pathological results suggest conCLNM. However, this approach has obvious limitations in clinical practice due to the unique structure of central lymph nodes and the difficult-to-control waiting time of frozen section pathological results during surgery. Although our study is retrospective in nature, this study not only included general clinical characteristics such as sex and age, which were easily obtained, combined with ultrasound characteristics such as tumor size and location on preoperative imaging, but also used the same immunological indicators that are more valuable in tumors, especially thyroid tumors, that can be conveniently obtained before surgery. All of these have greatly contributed to the clinical application of this approach. In recent years, several studies have shown that immune function, inflammation, and tumorigenesis are closely related. Ceylan et al. (14) found that both NLR and PLR are related to the aggressive biological behavior of PTC and can be used as biomarkers for the risk assessment of PTC patients. Meanwhile, Zhao et al. (23) discovered that the integration of the preoperative SII index and the tumor diameter is a reliable predictor of lymph node metastasis in patients with PTC. However, despite the inclusion of numerous inflammatory indicators, this study did not observe significant predictive effects of SII, NLR, and MLR in terms of conCLNM or conMVCLNM. This limitation may be attributed to the insufficient sample size, which failed to comprehensively elucidate the issue at hand, and thus necessitates future investigations with larger sample sizes. Furthermore, Huang et al. (24) demonstrated that the reduction of PLR poses a potential risk for recurrence in patients with intermediate and high-risk PTC. Similarly, Li et al. (25) conducted a comparable investigation and revealed that combining PLR and other inflammatory indicators with clinical characteristics significantly aids in predicting the recurrence risk of PTC. The current study also identified PLR as a risk factor for conCLNM or conMVCLNM, with a higher PLR ratio correlating with an increased likelihood of conCLNM. However, further research is required to elucidate the specific underlying mechanism.

In addition, in recent years, the definition of nodal volume metastasis has been revised to refer to the number of metastatic lymph nodes. For example, the meta-analysis of Wang et al. (26) defined high-volume lymph node metastasis in PTC as involving >5 lymph nodes. At the same time, Huang et al. (27) and Zhu et al. (28) have adopted the same definition of large-volume lymph node metastasis. However, the objective of these studies was confined to the quantification of metastatic lymph nodes, with only a few reports incorporating the assessment of metastatic lymph node diameter. Research has demonstrated that lymph node metastases >2 mm in diameter substantially impact the likelihood of recurrence in patients diagnosed with PTC (29). According to the guidelines established by the ATA, the risk of recurrence is notably elevated in PTC patients presenting with <5 metastatic lymph nodes that are >2 mm in diameter.

The definition of medium-volume metastatic lymph nodes, as presented in this study, aligns more closely with the clinical practice guidelines. Within our study cohort of 260 patients, 34 patients were identified as having conMVCLNM. Among these patients, 27 had conCLNM with <5 metastatic lesions, yet the diameter of the metastatic lesions >2 mm. By only considering the number of metastatic lymph nodes, physicians may overlook these cases, resulting in a notable 10.4% (27/260) increase in the risk of recurrence. Therefore, in contrast to previous studies, our research carries greater significance in informing and improving practical clinical practices. This is because our approach exhibits improved effectiveness in assessing the probability of conMVCLNM in patients with uPTC and provides more effective treatment insights to enhance their prognosis.

Furthermore, previous studies have indicated that the predictive accuracy of conCLNM and conMVCLNM is relatively low. This may be attributed to the limited efficacy of the direct diagnosis of CLNM in clinical practice. For example, Kim et al. (30) found that ultrasound had a sensitivity of only 17.3% in detecting CLNM, whereas computed tomography had a sensitivity of only 23.5%. Additionally, certain clinicopathological characteristics, including gender, age, and tumor lesion information, have been shown to be effective predictors of CLNM. Feng et al. (31) developed a prediction model for conCLNM in PTC by incorporating lesion location, ETE, and other factors. The model predicted an area under the curve (AUC) of 0.754 for conCLNM. By contrast, the AUC of this study was found to be 0.837 (95% CI 0.759–0.916), with a sensitivity of 78.8% and a specificity of 79.4%, which were significantly higher than previous results. Additionally, the prediction model offers a visual representation of the results, serving as a valuable clinical tool for thyroid surgeons in formulating surgical plans. Therefore, it is recommended for widespread adoption and implementation.

Our study has the following limitations. First, the sample size of this study is not large enough. In the future, we will further expand the sample size to improve the efficiency of predicting conMVCLNM. Second, this study is a retrospective study, and there is a certain selection bias in the sample selection. We hope that prospective studies will be conducted in the future. Third, preoperative immune indicators are affected by various factors, such as inflammation, immune system diseases, and other interferences. In the future, we will add more preoperative clinical pathology information, such as genetic diagnostic information, to effectively establish a predictive model for conMVCLNM before surgery.

In conclusion, our study provides an effective method for clinically identifying uPTC patients with conMVCLNM. When uPTC patient characteristics are combined with high-risk factors, such as age, gender, tumor diameter, and PLR, contralateral central lymph node dissection is strongly recommended.

## Data availability statement

The raw data supporting the conclusions of this article will be made available by the authors, without undue reservation.

## Ethics statement

This study was approved by Ethics Committee of Hangzhou First People’s Hospital and written informed consent was obtained from all patients.

## Author contributions

FW: Validation, Writing – original draft, Writing – review & editing. YT: Conceptualization, Data curation, Writing – review & editing. YZ: Investigation, Methodology, Supervision, Writing – review & editing. KH: Formal analysis, Project administration, Writing – review & editing. XH: Formal analysis, Project administration, Writing – review & editing. TP: Methodology, Supervision, Writing – original draft. JS: Data curation, Methodology, Writing – original draft. JD: Data curation, Formal analysis, Writing – review & editing. GP: Data curation, Methodology, Writing – review & editing. YP: Data curation, Formal analysis, Writing – review & editing. LZ: Investigation, Validation, Writing – review & editing. DL: Conceptualization, Data curation, Writing – original draft. YL: Validation, Writing – original draft, Writing – review & editing.
